# Case Report: Herceptin as a Potentially Valuable Adjuvant Therapy for a Patient With Human Epidermal Growth Factor Receptor 2-Positive Advanced Esophageal Squamous Cell Carcinoma

**DOI:** 10.3389/fonc.2020.600459

**Published:** 2021-02-01

**Authors:** Li Han, Chi Pan, Qingtao Ni, Tao Yu

**Affiliations:** ^1^ Department of Medical Oncology, Xuzhou first People’s Hospital, The Affiliated Xuzhou Municipal Hospital of Xuzhou Medical University, Xuzhou, China; ^2^ Department of General Surgery, Jiangsu Taizhou People’s Hospital, Taizhou, China; ^3^ Department of Oncology, Jiangsu Taizhou People’s Hospital, Taizhou, China

**Keywords:** esophageal squamous cell carcinoma, herceptin, chemotherapy, targeted therapy, human epidermal growth factor receptor 2 (Her2) positive

## Abstract

Esophageal cancer is one of the most common cancers with a low overall 5-year relative survival rate of approximately 20%. Trastuzumab (Herceptin^®^) targets HER2 and is an effective therapeutic strategy in HER2-positive breast cancer. However, few reports have described targeted therapy for treating esophageal squamous cell carcinoma (ESCC). A patient with advanced ESCC who had received chemotherapy, radiotherapy, and had undergone a clinical study is described here. The tumor had not been controlled. Herceptin and chemotherapy were used as salvage therapy in this patient because of high HER2 expression. Good therapeutic results were observed in this patient. Therefore, Herceptin is a potential target therapy for patients with HER2-positive advanced ESCC. A study with a large population and a prospective random study are necessary to validate these results.

## Highlights

Herceptin is a potential target therapy for patients with advanced HER2-positive esophageal squamous cell carcinoma.
HER2 gene testing should not be ignored in squamous cell carcinoma.

## Introduction

Esophageal cancer is one of the most common cancers worldwide. The low overall 5-year relative survival rate of patients with esophageal cancer of approximately 20% is one of the lowest of all cancers (1). Therefore, it is urgent and necessary to identify novel treatment strategies in esophageal cancer. The two major esophageal cancer subtypes are esophageal squamous cell carcinoma (ESCC) and esophageal adenocarcinoma (EAC) (2). With the development of precision medicine, targeted therapy is becoming one of the most promising approaches for treating all cancers. Human epidermal growth factor receptor 2 (HER2) is overexpressed in 20–30% of breast cancers (3, 4). HER2 over-expression leads to abnormal cell proliferation and results in poor prognosis in HER2-positive breast cancer (5). HER2 overexpression is also observed in primary squamous cell carcinoma of the breast (6). Apart from breast, gastric, and gastroesophageal cancers, HER2 amplification of about 2% is observed in multiple tumors (7). Trastuzumab (Herceptin^®^), targets HER2 and is an effective therapeutic strategy in HER2-positive breast cancer (4). However, there have been few reports exploring targeted therapy in esophageal squamous cell carcinoma (ESCC). We reported a case of HER2-positive advanced ESCC that was treated using Herceptin and chemotherapy with good therapeutic results in our department.

## Case Report

A 61-year-old man was referred to our hospital with dysphagia in November 2017. Gastroscopy showed that two new growths were observed in his esophageal mucosa. One was an 18–22 cm growth originating from his incisor, and the other was a 25–40 cm ring wall growth. Pathology results showed that the new tumors were squamous cell carcinoma and the diagnosis was cervical and lower thoracic esophageal carcinoma. Surgery was not an option for this patient because of the two long lesions in esophagus. Therefore, two cycles of chemotherapy were administered to the patient using “paclitaxel liposome 240 mg (135 mg/m^2^) + nedaplatin 100 mg (55 mg/m^2^)” in December 2017. The curative effect was evaluated by partial remission (PR). From January 2 to March 1, 2018, the patient received radical radiotherapy with DT50.4GY/28F. The patient also received concurrent chemotherapy with “paclitaxel liposome 240 mg + nedaplatin 100 mg” on January 22 2018. However, lung computed tomography (CT) on June 27, 2018 showed stage IV lung metastasis. Meanwhile, dysphagia was not relieved. Subsequently, the patient was enrolled in the “BGB-A317-302” clinical study in July 2018. “BGB-A317-302” is a randomized, controlled, open, global phase three study, comparing the efficacy of the BGB-A317 anti-PD-1 antibody with chemotherapy as a second-line treatment in patients with advanced esophageal cancer. Two cycles of trial drug treatment were performed on July 23, 2018 and August 13, 2018. Unfortunately, head skin metastasis was identified on August 22, 2018. The curative effect was evaluated as progressive disease (PD), and the patient was excluded from the study.

**Figure 1:**
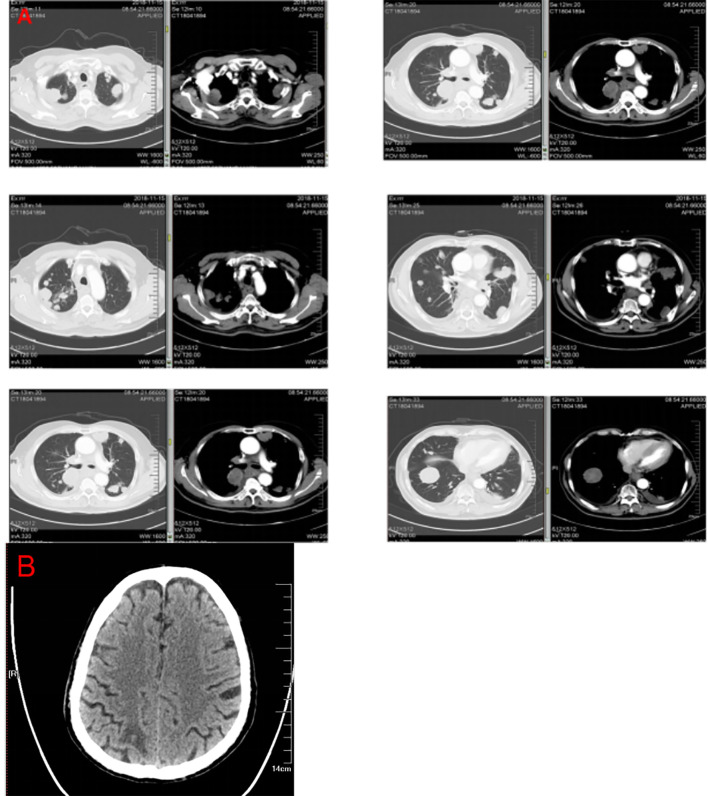
Computed tomography images from the patient with esophageal squamous cell carcinoma. Computed tomography imaging revealed an expansile intraluminal esophageal mass and lung masses **(A)**. The mass observed in brain computed tomography **(B)**.

**Figure 2:**
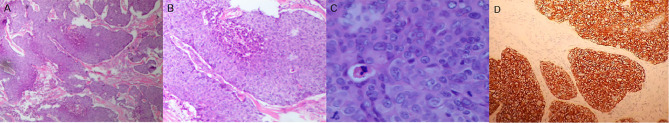
The mass on the chest wall was composed of poorly differentiated mucinous adenocarcinoma of the solid type. **(A)** Hematoxylin and eosin (H&E) staining, ×40. **(B)** H&E staining, ×200. **(C)** H&E staining, ×400. **(D)** Her2 staining, ×200. H&E, hematoxylin and eosin.

Two months later, the patient had increasing lymphadenopathy in the left supraclavicular mass enlargement with skin ulceration. On November 14 2018, he came to our hospital for further treatment of the left supraclavicular mass. Laboratory tests showed carbohydrate antigen 125 (CA125): 106.1 µ/ml, neuron-specific enolase (NSE): 32.59 µ/ml, CYFR-211: 89.00 ng/ml. The masses were visualized by chest and head CT (**Figure 1**). Resection of the left supraclavicular mass was performed on November 15 2018. Postoperative pathology showed that squamous cell carcinoma was moderately differentiated, with ulcer formation and invasion of striated muscle tissue (**Figures 2A–C**). It was considered as metastasis based on medical history (IV stage).

**Figure 3:**
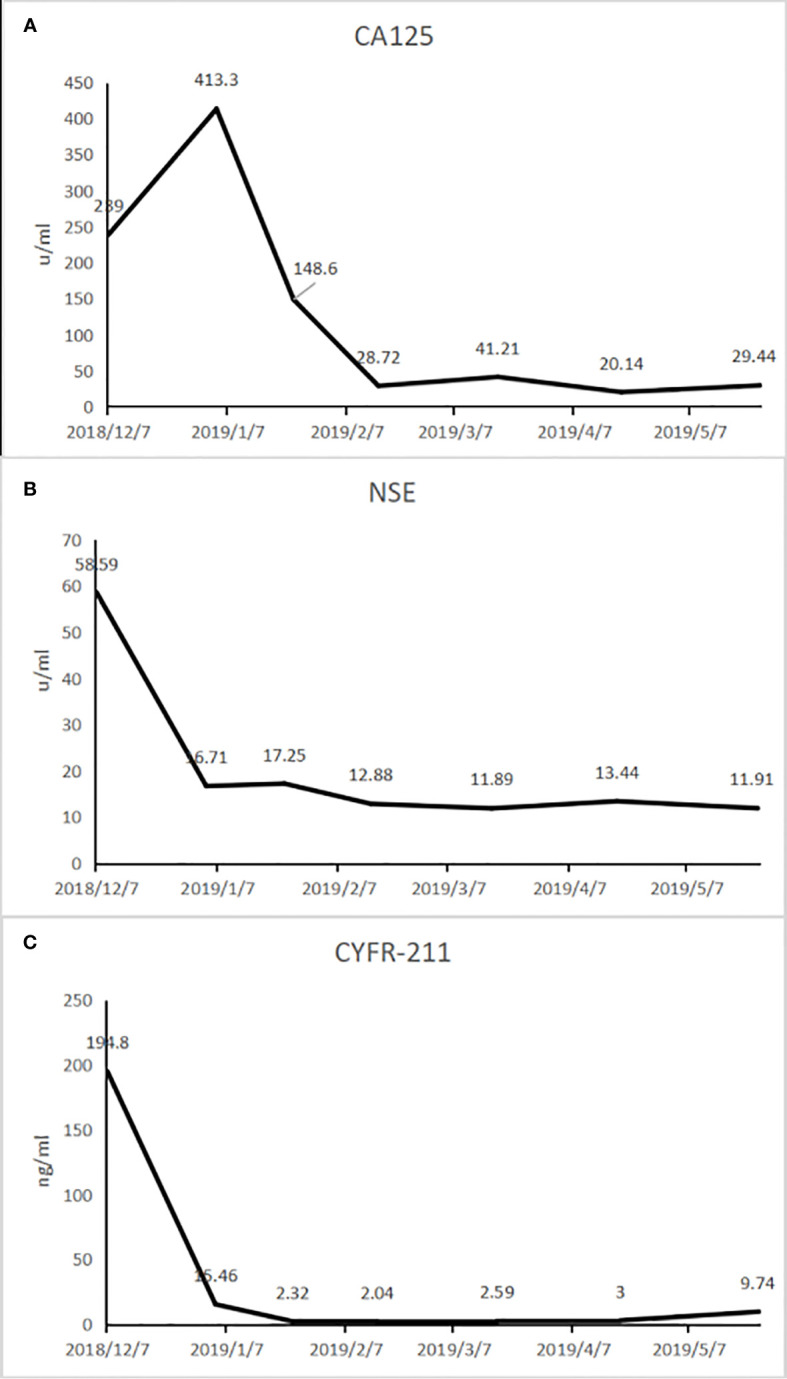
The tumor markers after treatment with Herceptin combined with chemotherapy. CA125 **(A)**, NSA **(B)**, and CYFR-211 **(C)** decreased after treatment with Herceptin combined with chemotherapy.

The patient had a strong desire for survival and his intervention adherence was high. Larotrecinib was approved by the Food and Drug Administration for the treatment of NTRK (Neuro Trophin Receptor Kinase) gene carriers on November 26 2018. Moreover, larotrecinib is safe, well tolerated, and a highly effective treatment for all NTRK positive cancer patients (8). Therefore, to assess future treatment options for esophageal carcinoma, tissue from the left supraclavicular mass of this patient was subjected to gene testing (Beijin Geneis Diag). This analysis revealed that the tumor sample was EGFR (epidermal growth factor receptor)-, HER2+ (>1%), NTRK-, and had MSS (Microsatellite stabilization). The immunohistological staining result showed HER2 (3+) (**Figure 2D**). It showed that larotrecinib was not a suitable treatment option. However, trastuzumab, the anti-HER2 directed therapy, can produce therapeutic benefits for HER2-positive patients. Laboratory tests results showed: carbohydrate antigen 125 (CA125), 239 µ/ml; neuron-specific enolase (NSE), 58.59 µ/ml; CYFR-211, 194.8 ng/ml (**Figure 3**).

**Figure 4:**
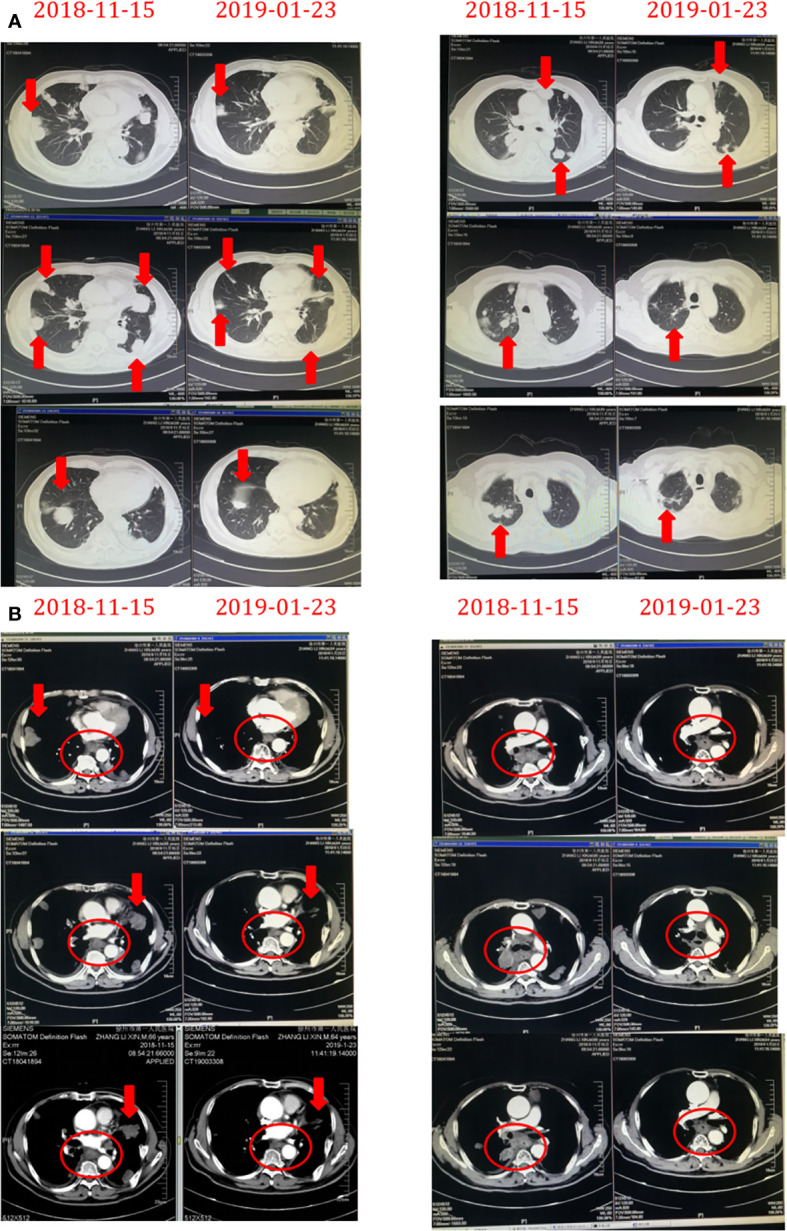
The chest masses before and after treatment with Herceptin combined with chemotherapy. The masses rapidly decreased in size on aortopulmonary window images **(A)** and mediastinum window images **(B)** derived by chest computed tomography after treatment with Herceptin combined with chemotherapy.

Therefore, 400 mg of (230 mg/m^2^) albumin bound paclitaxel, 90 mg of (50 mg/m^2^) cisplatin, and 420 mg of (6 mg/kg) trastuzumab (Herceptin) were administered to the patient on December 10, 2018 and January 04, 2019 as the first-line anti-HER-2 drug treatment. Chest CT re-examination on January 23, 2019 showed that the mass was significantly reduced in size (**Figure 4**). Tumor biomarkers gradually decreased (**Figure 3**). PR was evaluated according using RECIST1.1 (9) and the esophageal obstruction was relieved. Treatment was well tolerated by the patient without any discomfort. The patient was again treated with the above regimen on January 25 and February 16. The effect of this treatment was reviewed and evaluated every two cycles and the patient was still evaluated by PR status.

**Figure 5:**
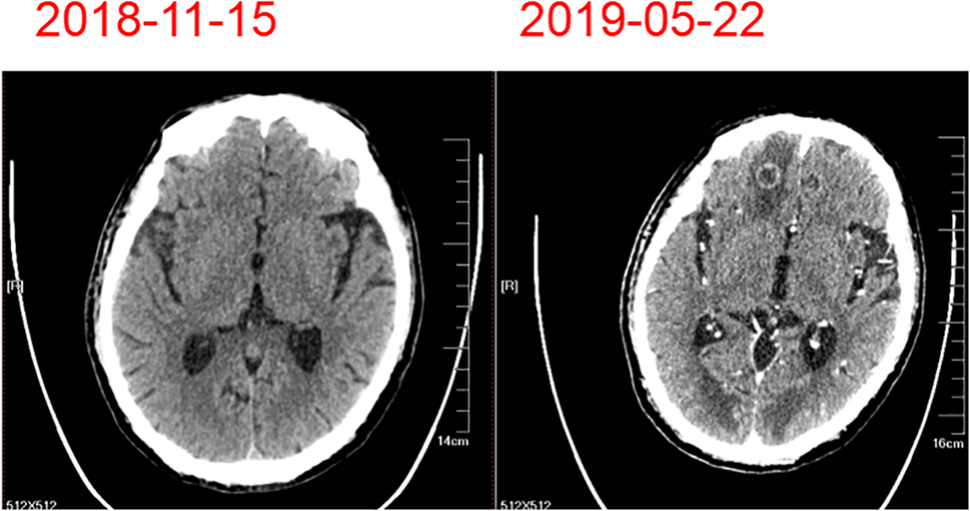
Brain masses observed by brain computed tomography before and after treatment.

**Figure 6:**
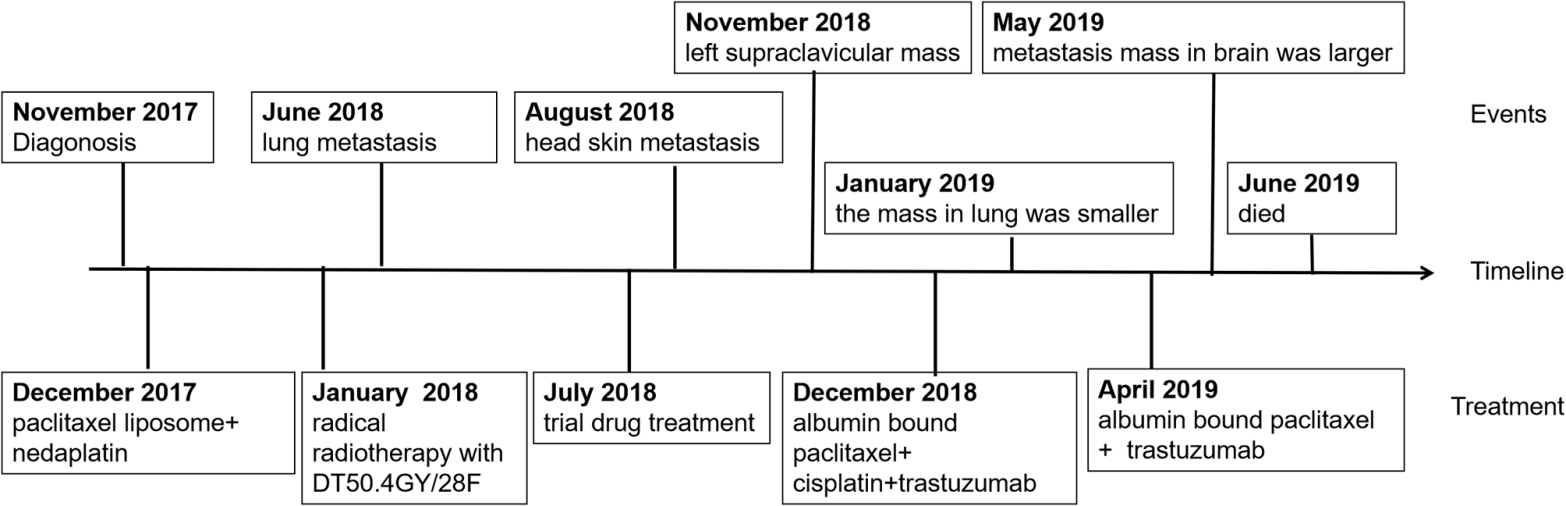
Timeline of events since diagnosis and summary of administered treatments.

On February 23, 2019, the patient developed grade IV myelosuppression. Peripheral blood count results were: white blood cell count, 1.2 × 10^9^/liter; red blood cell count, 2.11 × 10^9^/liter; hemoglobin 66 g/liter; and platelet count, 64 × 10^9^/liter. After supportive transfusions of two units of red blood cells over two days, 200 µg granulocyte colony stimulating factor, and 3 mg of interleukin-6 for four days, the patient’s condition quickly improved. Subsequently, following the insistence of the patient, one more cycle of the anti-HER-2 drug treatment was administered on March 21. However, the patient developed numbness in both lower limbs which was considered a side effect caused by cisplatin. Therefore, cisplatin was discontinued and only 400 mg of albumin bound paclitaxel and 420 mg of trastuzumab (Herceptin) were administered on April 26, 2019. The patient was in good clinical status with complete remission of dysphagia until May 19, 2019. At this time, the patient experienced dizziness and unstable walking. The metastatic mass was larger than before and new multiple lesions were found in brain CT (**Figure 5**). Whole brain radiotherapy was recommended to mitigate neurological symptoms. However, the patient refused further treatment and died of brain metastases in June 2019. This patient had over 6 months progression-free survival (PFS) and an overall survival (OS) of 19 months. This patient’s diagnosis and treatment are summarized in **Figure 6**.

## Discussion

Esophageal cancer is one of the most common malignant cancers in China (10). Drinking hot tea may be an important risk for esophageal cancer (11). We reported a patient with advanced ESCC with brain, lung, and skin metastases. The optimal treatment course for patients with advanced ESCC remains controversial (12). Comprehensive treatment (chemotherapy and radiotherapy) is the only treatment with potentially curative intent in patients with advanced ESCC (13). Even so, advanced ESCC still has poor prognosis and a low survival rate. Therefore, there remains a need for the development of effective treatment. Herceptin, a recombinant monoclonal antibody to HER2, is as an effective drug for improving outcomes in patients with HER2-positive breast cancer (14). Other adenocarcinomas also have high HER2 expression. For example, HER2 is also overexpressed in approximately 20% of gastric and esophagogastric junction adenocarcinoma (15). HER2 expression and amplification is common in esophageal adenocarcinoma (2). HER2 amplification is observed in 17% of esophageal adenocarcinoma (16).

Trastuzumab combined with chemotherapy is recommended as a new therapy for patients with HER2-positive advanced gastric and esophagogastric junction adenocarcinoma (17). This treatment improved the median OS by 2.7 months in patients with HER2-positive gastric/gastroesophageal junction cancer compared with chemotherapy alone (18). Additionally, HER2-targeted therapies were recently updated in the NCCN Guidelines to a category 2A recommendation in rectal cancer (19). To date, there are no reports of HER2-targeted therapies in esophageal cancer. This is the first report of a patient with HER2-positive advanced ESCC being treated with Herceptin and achieving clinical remission. It is a pity that neurological symptoms appeared following the progression of brain metastases 6 months after successful treatment. We postulate that this may be caused by many different reasons. Firstly, discontinuation of cisplatin in the sixth chemotherapy course because of myelosuppression could be a major element. Previous research has indicated clinical synergy between cisplatin and trastuzumab (20). Cisplatin discontinuation may reduce Herceptin efficacy. Secondly, resistance to Herceptin may contribute to cancer progression. The mechanism of HER2-targeted resistance remains unknown. At present, many potential pathways are involved in HER2-targeted resistance, including cross-talk with estrogen receptors, immune response, and cell cycle control mechanisms (21). MyPathway, a phase IIa multiple basket study, indicated that 38% of patients with colorectal cancer with HER2 amplification achieved PR after treatment with patuzumab combined with the HER2 inhibitor trastuzumab (22). Mengjun et al. found the GB235 anti-HER2 antibody can be considered a complementary therapy for HER2-positive breast cancer (23). Therefore, it is necessary to formulate complementary therapy for HER2-positive patients. Lastly, the blood brain barrier may contribute to brain metastasis. Indeed, the concentration of Herceptin in cerebrospinal fluid is only 0.5% that measured in blood (24). While the mechanism of this is not clear, it may be related to the difficulty of macromolecules passing through the blood-brain barrier.

We reported a patient with HER2-positive ESCC who received trastuzumab combined with chemotherapy and who experienced over 6 months PFS and 19 months OS. In clinical practice observation, patients with gastroesophageal or gastric metastatic adenocarcinoma treated with trastuzumab had 7.9 months PFS and 14.1 months OS (25). This suggests that the effect of Herceptin treatment is likely similar in HER2-positive patients with ESCC and those with adenocarcinoma. However, the response rate of Herceptin treatment in all patients with HER2-positive ESCC remains unknown and needs to be determined in a large population.

## Conclusion

Herceptin is a potential therapy for patients with advanced HER2-positive ESCC. Therefore, HER2-positive gene tests should not be ignored in squamous cell carcinoma. This conclusion needs validation through prospective random and large population studies.

## Data Availability Statement

The original contributions presented in the study are included in the article/supplementary materials; further inquiries can be directed to the corresponding author.

## Ethics Statement

The study was approved by the Human Ethics Review Committee of Xuzhou first People’s Hospital. Written informed consent was obtained from the individual(s) for the publication of any potentially identifiable images or data included in this article.

## Author Contributions

LH and TY provided this case. LH, QN, and CP wrote, reviewed, and/or modified the manuscript. All authors contributed to the article and approved the submitted version.

## Conflict of Interest

The authors declare that the research was conducted in the absence of any commercial or financial relationships that could be construed as a potential conflict of interest.
